# The antibacterial effect of chlorohexidine, Er:YAG laser and diode laser 980 nm as dental cavity disinfectants of dentine: an in vitro study

**DOI:** 10.1186/s40001-025-03595-z

**Published:** 2025-12-29

**Authors:** Mennatallah Khafagi, Mostafa Gheith, Haythem S. Moharrum, Mohamed Abo Elyazeed Ahmed, Riham M. Aly, Ahmed A. Hamed, Maryam El Mansy

**Affiliations:** 1 Pediatric Dentistry Orthodontics and Pediatric Dentistry Department, Oral and Dental Research Institute, National Research Centre, 33 El Buhouth St, Dokki, Cairo, Egypt; 2 Laser Applications in Dental Medicine, National Institute of Laser Enhanced Sciences, Cairo University, Giza, Egypt; 3 Department of Medical Applications of Laser, National Institute of Laser Enhanced Sciences, Cairo University, Giza, Egypt; 4 Stem Cell Laboratory, Center of Excellence for Advanced Sciences, National Research Centre, Cairo, Egypt; 5 Department of Basic Dental Science, Oral Medicine & Dentistry Research Institute, National Research Centre, 33 El Buhouth St., Dokki, Cairo, 12622 Egypt; 6 Microbial Chemistry Department, National Research Centre, 33 El-Buhouth Street, Dokki, Giza, 12622 Egypt

**Keywords:** Cavity disinfection, Diode laser, Erbium YAG laser, *Streptococcus mutans*

## Abstract

**Objectives:**

Comparison between different disinfection modalities in reducing *Streptococcus mutans* count within dental cavities. Research question: whether using an antibacterial agent has a value in reducing the bacterial load in the dental cavity and what is the best modality.

Cariogenic bacteria can remain viable in the dentine even after complete removal of caries, so the use of cavity disinfectants before restoration placement is crucial, such as chlorohexidine, Er:YAG and Diode laser 980 nm. The aim of this study was to evaluate and compare the antibacterial effect of chlorohexidine, Er:YAG and diode laser 980 nm as cavity disinfectants.

**Methods:**

Forty sound extracted human primary molars were randomly assigned to four groups (ten teeth in each group); group I: (negative control): no disinfection was applied, group II: (positive control): application of 2% chlorhexidine gluconate solution, group III: a 980 nm diode laser (LASOTRONIX, Poland) was used at 1 W in continuous wave mode, and group IV: an Er:YAG laser was used at a, power 1.2 W. The antibacterial efficacy against *Streptococcus mutans* bacteria was assessed via colony forming units (CFU) and optical density of bacteria before treatment and after treatment.

**Results:**

A statistically significant increase in bacterial count was observed in the negative control group (Gp1). Treatment groups 2, 3, and 4 showed a significant reduction in bacterial count from baseline to post-treatment.Er:YAG group showed the highest percentage of reduction of bacterial count with a statistically significant difference with other groups. No statistically significant difference was shown between chlorohexidine and diode laser groups.

**Conclusions:**

The Er:YAG laser demonstrated the highest antibacterial efficacy against *Streptococcus mutans* when compared with chlorhexidine and the diode laser (980 nm). Although all three disinfection methods showed significant antibacterial activity.

*Clinical relevance*: The superior antibacterial performance of the Er:YAG laser supports its use in clinical practice for managing deep carious lesions. These findings highlight the importance of thorough cavity disinfection before restoration placement to minimize bacterial persistence and reduce the risk of secondary caries. Clinical trial number: not applicable.

**Supplementary Information:**

The online version contains supplementary material available at 10.1186/s40001-025-03595-z.

## Introduction

Dental caries is a widespread, multifactorial disease caused by the interaction of bacteria, dietary sugars, and host factors. It begins with biofilm formation on teeth, where acid-producing bacteria metabolizes sugars to produce acids. These acids demineralize tooth enamel and other hard tissues, leading to tooth caries [1].

Although complete removal of caries and necrotic tissue is crucial for the clinical success of restoration, cariogenic bacterial could be pushed deeper into dentinal tubules during the process of caries removal, where they can be viable for extended periods.this could be one of the main causes of secondary caries [2]. Bacteria within the smear layer can remain active and multiply, with their metabolic by-products potentially reaching the dental pulp and triggering inflammation. In addition, bacteria may infiltrate between restoration tooth interfaces leading to microleakage which is the main reason of restoration failure [3].

One of the most causative micro-organisms of primary and secondary caries is the *Streptococcus mutans* which is widely acknowledged as the primary bacterium responsible for dental caries [4]. It has a high cariogenic potential due to its ability to ferment dietary sugars into lactic acid which demineralize the tooth and degrade restorative materials, causing gaps and microleakage that lead to caries recurrence. Over time, this biofilm can further weaken the bond between the tooth and the restorative material, accelerating the restoration’s failure [5].

Preventing secondary caries at the dentine–restoration interface is vital in restorative dentistry. Disinfection methods aim to eliminate *Streptococcus mutans* using agents, such as chlorhexidine, ozone, and silver diamine fluoride and laser [6] Chlorhexidine which is the gold standard due to its strong and lasting antimicrobial action. Recently, lasers have emerged as effective tools for cavity disinfection, offering deep penetration, minimal tissue damage, and enhanced dentin bonding, making them valuable in modern restorative care [7].

Despite of its advantages, CHX has some drawbacks; it has a limited penetration depth in deep cavities and may not effectively reach deeper layers of infected dentin, limiting its effectiveness in cases, where the bacteria have penetrated further into the tooth structure. Furthermore, it may cause staining of teeth and alteration of taste in some cases [8].

Lasers have gained popularity in dentistry due to their versatile applications, with one significant use being the disinfection of the dentine in both root canal system and dental cavities [9] Research indicates that lasers can penetrate deeper than 400 μm into the dentinal tubules, effectively eliminating bacteria at these depths [10].

In addition to their bactericidal effects, lasers can occlude dentinal tubules, effectively sealing off pathways for bacterial reinfection. The antibacterial action of lasers on target cells, tissues, or organisms may involve photochemical interactions from both soft and hard lasers, while hard lasers can also produce photo-thermal, photo-ablative, or photo-mechanical effects [11].

Different types of lasers exhibit varying antibacterial effects on different micro-organisms. For instance, diode and erbium lasers can effectively kill bacteria and remove residual caries without causing thermal damage to the surrounding healthy tooth structure [12].

Diode laser has recently gained popularity and become widely used in dental clinics due to its affordability, portability, and effective bactericidal action achieved through thermal effects while maintaining a temperature rise that remains safe for permanent teeth. [13]**.** Erbium Laser works through the absorption of energy by water in bacteria dental tissues, which leads to the rapid vaporization of caries [14]. This method not only cleans cavity walls more effectively than traditional techniques but also exhibits a bactericidal effect by generating heat that can eliminate micro-organisms in the treated area with minimal thermal damage to surrounding healthy tissues which ensures a safer procedure [15]**.**

**Fig. 1:**
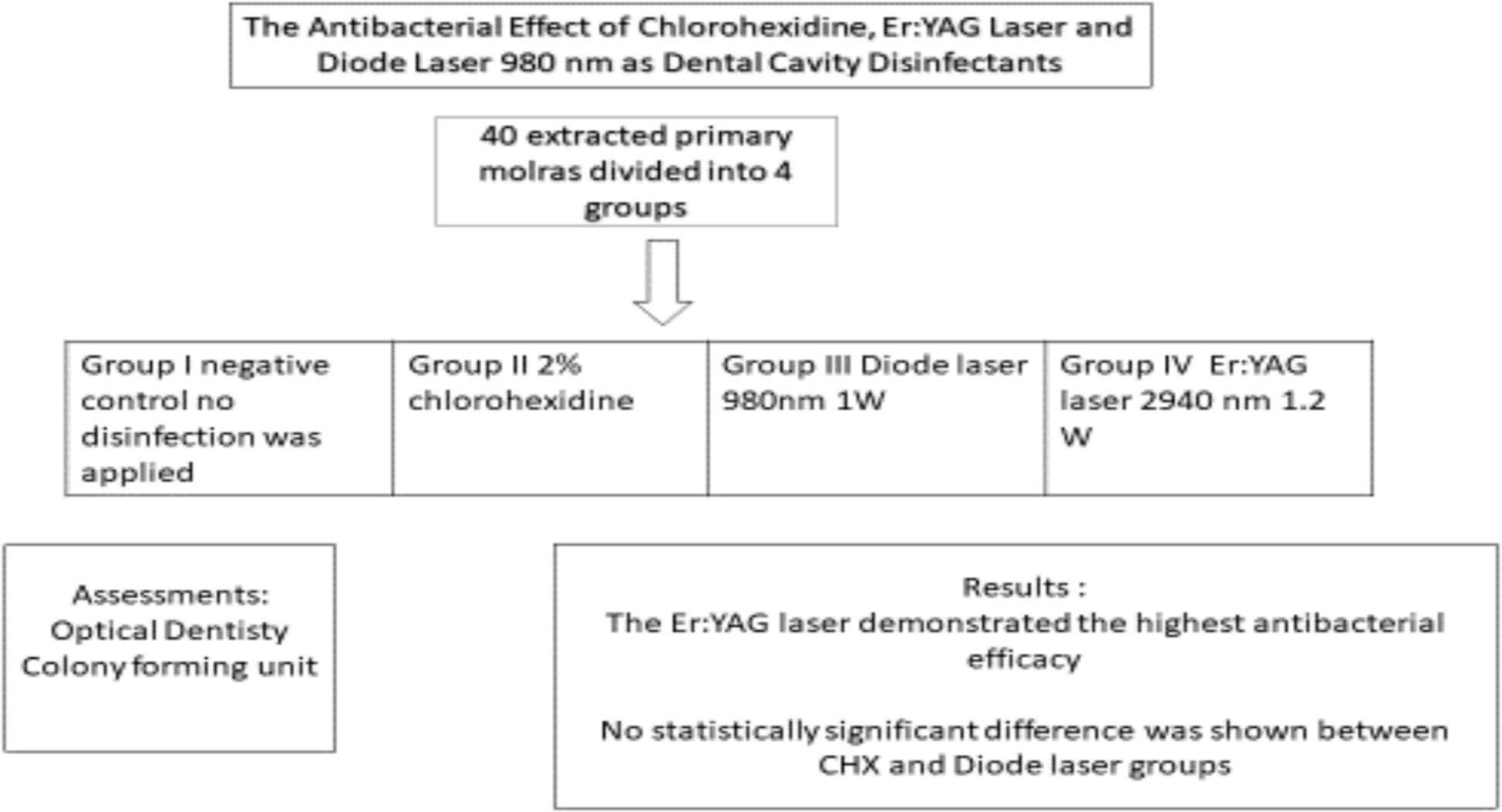
presents the schematic abstract of the study

This study was conducted to compare the antimicrobial efficacy of chlorhexidine, diode laser, and Er:YAG laser in reducing *Streptococcus mutans* counts within prepared dental cavities. The central hypothesis is that laser-assisted cavity disinfection, particularly using the Er:YAG laser, will result in a significantly greater reduction in *S. mutans* compared to traditional chlorhexidine treatment (Fig. 1). By evaluating and comparing these methods, this research aims to identify the most effective disinfection strategy to enhance restoration success and reduce the risk of secondary caries.

## Materials and methods

### Study design

This in vitro experimental study followed the CRIS Guidelines (Checklist for Reporting In-vitro Studies) published in 2014 to ensure the quality and transparency of experimental dental research [16]**.** A total of 40 freshly extracted human primary molars were utilized. The teeth were extracted at or near their natural exfoliation period to minimize variability in tooth structure and health status.

### Ethical considerations

The authors assume full responsibility for all elements of the study, ensuring that any concerns regarding the accuracy or integrity of any component are thoroughly examined and appropriately addressed. All procedures were conducted in strict adherence to the ethical principles outlined in the Declaration of Helsinki. Informed consent was obtained from all individuals prior to their participation. The experimental protocol received ethical approval from the Medical Research Ethics Committee of the National Research (approval no1234052022.). On 7/4/2022.

### Sample size calculation

The sample size calculation was based on the study by Jamel and Taher (2024), who evaluated the antibacterial efficacy of a 940 nm diode laser against *Streptococcus mutans*. In their study, the mean ± standard deviation of CFU counts was 34 ± 5.8 in Group I and 25 ± 3.6 in Group II, corresponding to an effect size of 1.86 with a power of 0.9 and a Type I error probability of 0.05. Based on these parameters, the minimum required sample size for the present study was calculated to be 8 cases. To account for a potential 20% dropout rate, the total sample size was increased to 10. The sample size calculation was performed using a *t* test in G*Power version 3.1.4.9. [17].

### Randomization and allocation

**Fig. 2:**
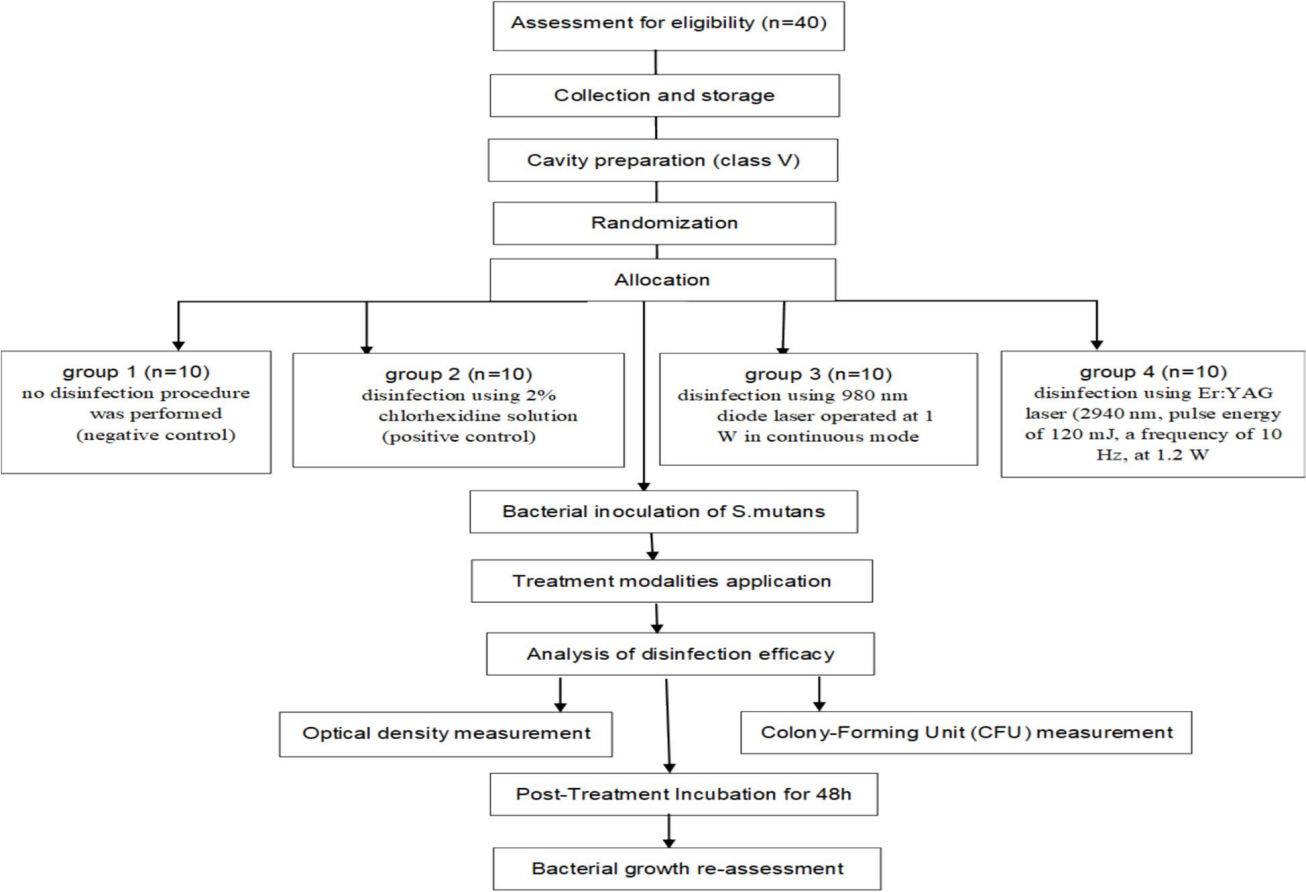
presents a schematic diagram summarizing the enrollment, randomization, allocation, treatment, and analysis of the samples

The 40 extracted molars were labeled from 1 to 40 and placed in a sealed, opaque, sterile container. Randomization into four equal groups (*n* = 10 per group) was performed using a random sequence generated on www.random.org on November 12, 2024, following a 1:1 allocation ratio (Fig. 2)

### Blinding

A double-blind protocol was followed. The microbiologist performing the bacterial assessments and the statistician conducting the data analysis were both blinded to the treatment assignments of the specimens.

### Selection and preparation of specimens

#### Inclusion and exclusion criteria

The inclusion criteria for this study comprised freshly extracted primary molars that were clinically sound and retained at least half of their root structure. In contrast, the exclusion criteria included teeth presenting with cracks, carious lesions, previous restorations, pulpal involvement, advanced structural destruction due to caries, developmental anomalies, or any history of prior restorative procedures.

#### Collection and storage

Teeth were collected from the outpatient pediatric dentistry clinic of the NRC. Parental consent was obtained prior to tooth extraction. They were informed about the study that was performed on their children’s teeth. After extraction, teeth were rinsed, cleaned of debris, and stored in deionized water at room temperature until further processing [18].

#### Cavity preparation

Standardized Class V cavities were prepared on the buccal surface of each tooth using a high-speed handpiece and a diamond bur (Horico Diament, Germany) under constant water cooling. The cavity dimensions were maintained at 3 mm mesiodistally × 2 mm occlusogingivally × 1.5 mm in depth, with the occlusal margin positioned 1 mm above the cementoenamel junction . A digital caliper was used to verify and ensure uniformity. A new bur was used after every five preparations [19]. Sterilization of teeth was achieved by autoclaving at 121c for 15 min. Each tooth was kept in a separate sterile sealed test tube [20].

### Experimental grouping

The samples were randomly allocated into four experimental groups. Group 1 served as the negative control, in which no disinfection procedure was performed. Group 2, designated as the positive control, received a 2% chlorhexidine gluconate solution (Grace for dental industries, Egypt) with 10 mL/min flow rate for 60 s [21]. In Group 3, disinfection was carried out using a 980 nm diode laser (LASOTRONIX, Poland) operated at 1 W in continuous mode [2]. Group 4 was treated with an Er:YAG laser (2940 nm, Fotona, Slovenia), set at a pulse energy of 120 mJ, a frequency of 10 Hz, and a total power output of 1.2 W [22].

### *Streptococcus mutans* culture

The identity of the isolation was confirmed through PCR-based genetic characterization following DNA extraction, utilizing species-specific genetic markers to ensure accurate identification. For culture preparation, a single bacterial colony was inoculated into Brain Heart Infusion (BHI) broth (Sigma-Aldrich) and incubated at 37 °C for 24 h. The resulting bacterial suspension was adjusted to a final concentration of 5 × 10^6^ CFU/mL. This concentration was verified by performing serial dilutions and plating on BHI agar to confirm the viable bacterial count.

### Antibacterial assessment protocol

#### Bacterial inoculation

Cavities in all samples were inoculated with the S. mutant’s suspension and incubated at 37 °C for 72 h to allow biofilm formation. The cavities were restored with a temporary filling (Cavit GC). After incubation period, the temporary filling was removed with an excavator then the dentin chips (20 + _ 5 mg) were collected from the internal walls of the cavities using a new sterile steel bur mounted to low speed contra angle hand piece. For every cavity a new sterile bur was used to avoid overheating of dentinal walls. The bur loaded with dentin chips was placed into sterile tubes containing 0.5 ml BHI mixed for 30 s to enable micro-organisms to pass through solution and produce a constant suspension [23].

- A) Optical density measurement

**Fig. 3:**
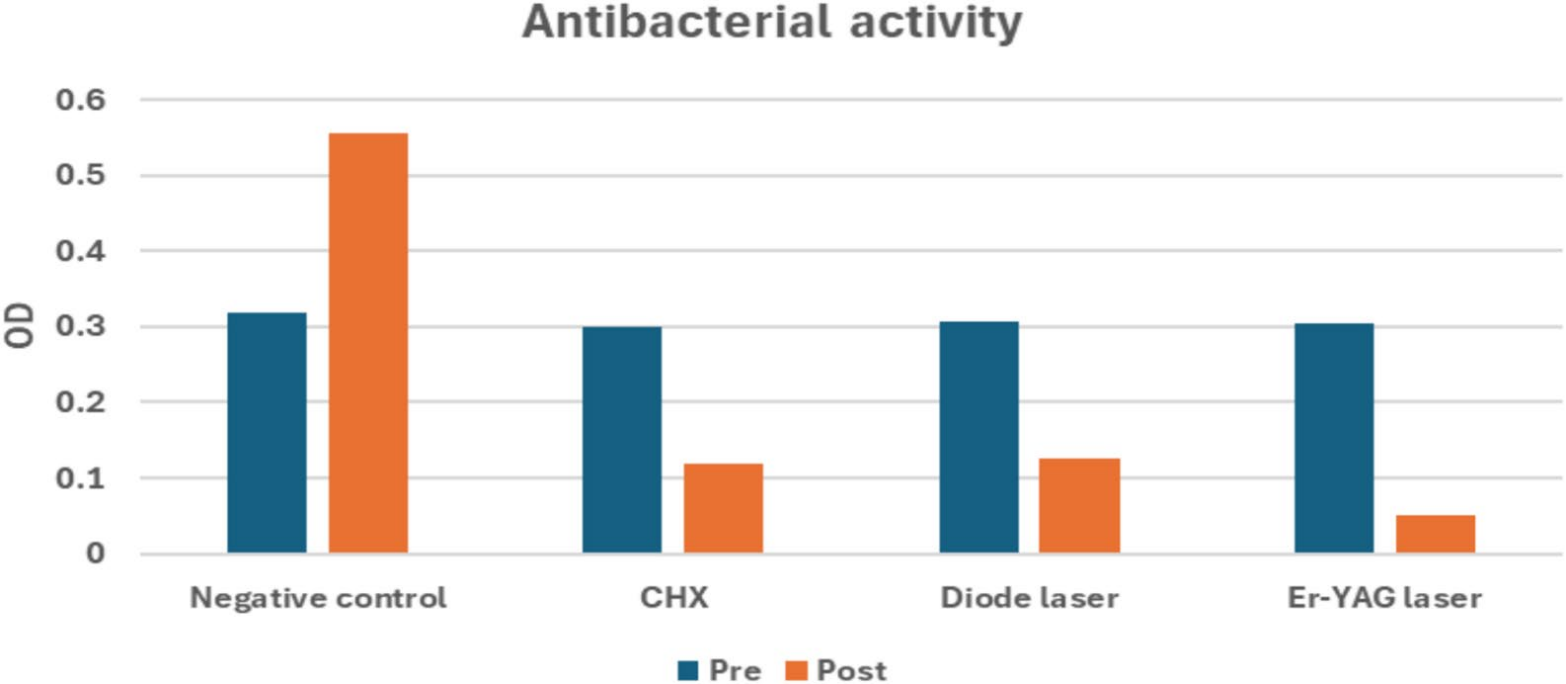
Bar chart representing antibacterial activity for different groups. Post-treatment comparison among groups revealed a significant difference (*p* < 0.001). The Er:YAG group had the lowest bacterial count, significantly lower than CHX and diode laser (*p* = 0.002 and *p* = 0.001), while no significant difference was found between CHX and diode laser, where (*p *= 0.970)

**Fig. 4:**
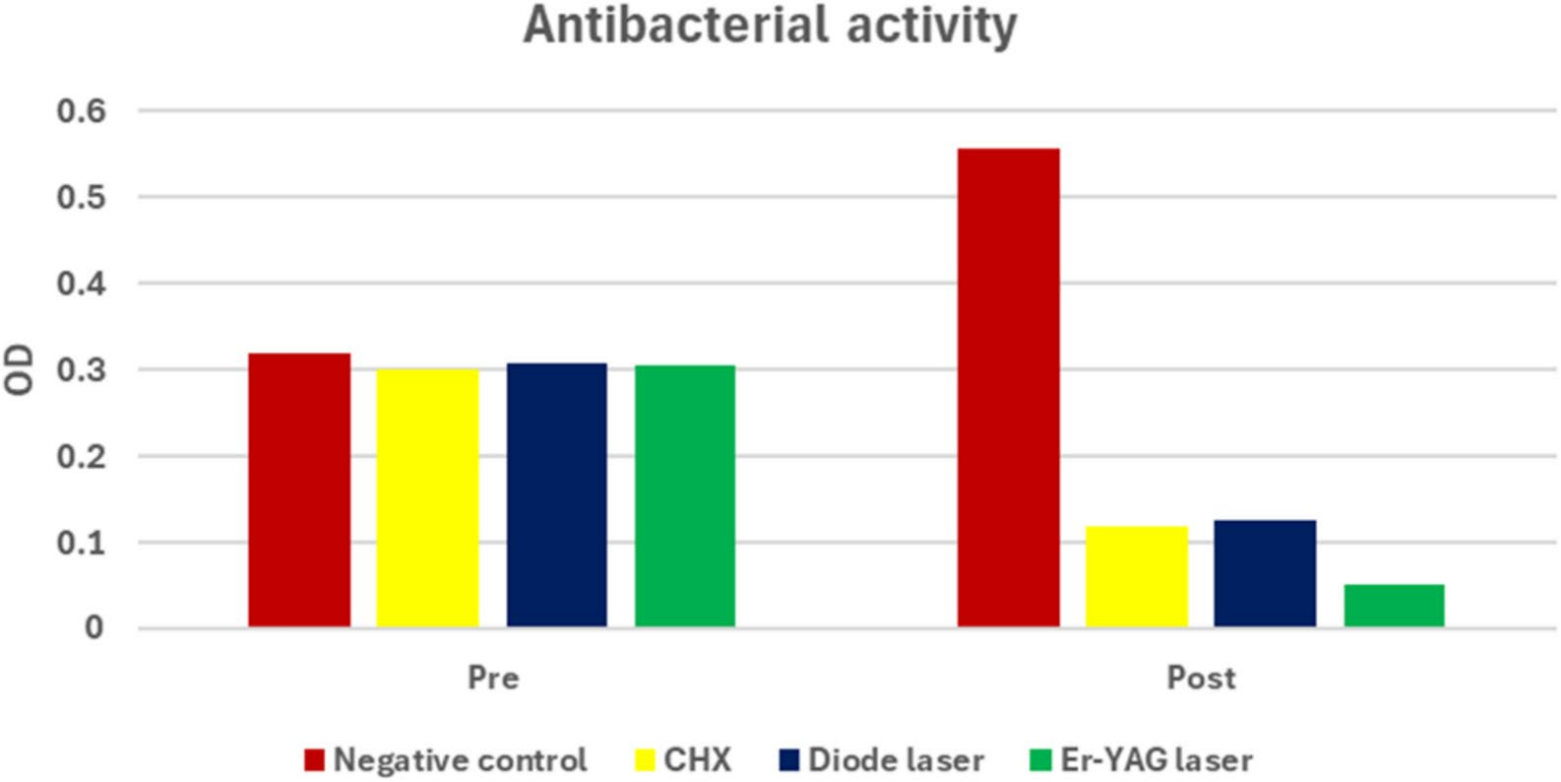
Bar chart representing antibacterial activity for different groups. Percentage of change analysis confirmed these findings: Er:YAG achieved 83.5% ± 4.08, CHX 60.2% ± 6.57, and diode laser 58.5% ± 6.84, compared with a bacterial increase of + 42.3% ± 8.09 in the negative control group

**Fig. 5:**
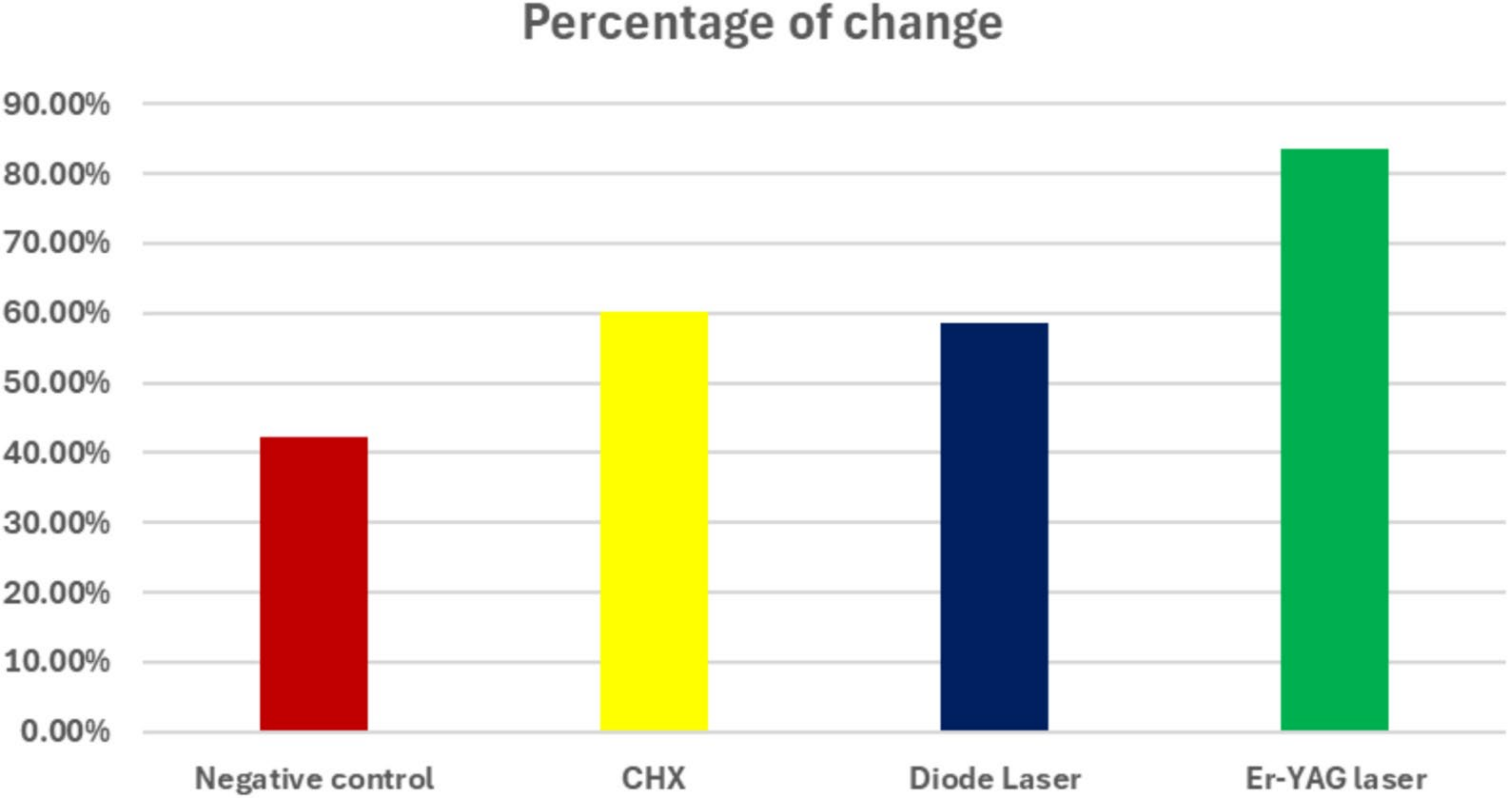
Bar chart representing percentage of bacterial change in different groups

50 µl was aspirated with an automatic pipette and placed in a 96-well polystyrene microplate. Baseline bacterial growth (pre-treatment reading) was recorded. A NanoStar microplate ELISA reader was employed to record absorbance values at appropriate wavelengths (600 nm) using 96 microplate polystyrene plate. Absorbance readings provided a quantitative assessment of bacterial load [24, 25].as shown in (Fig. 3, 4, 5)

- B) Colony-forming unit (CFU) measurement

**Fig. 6:**
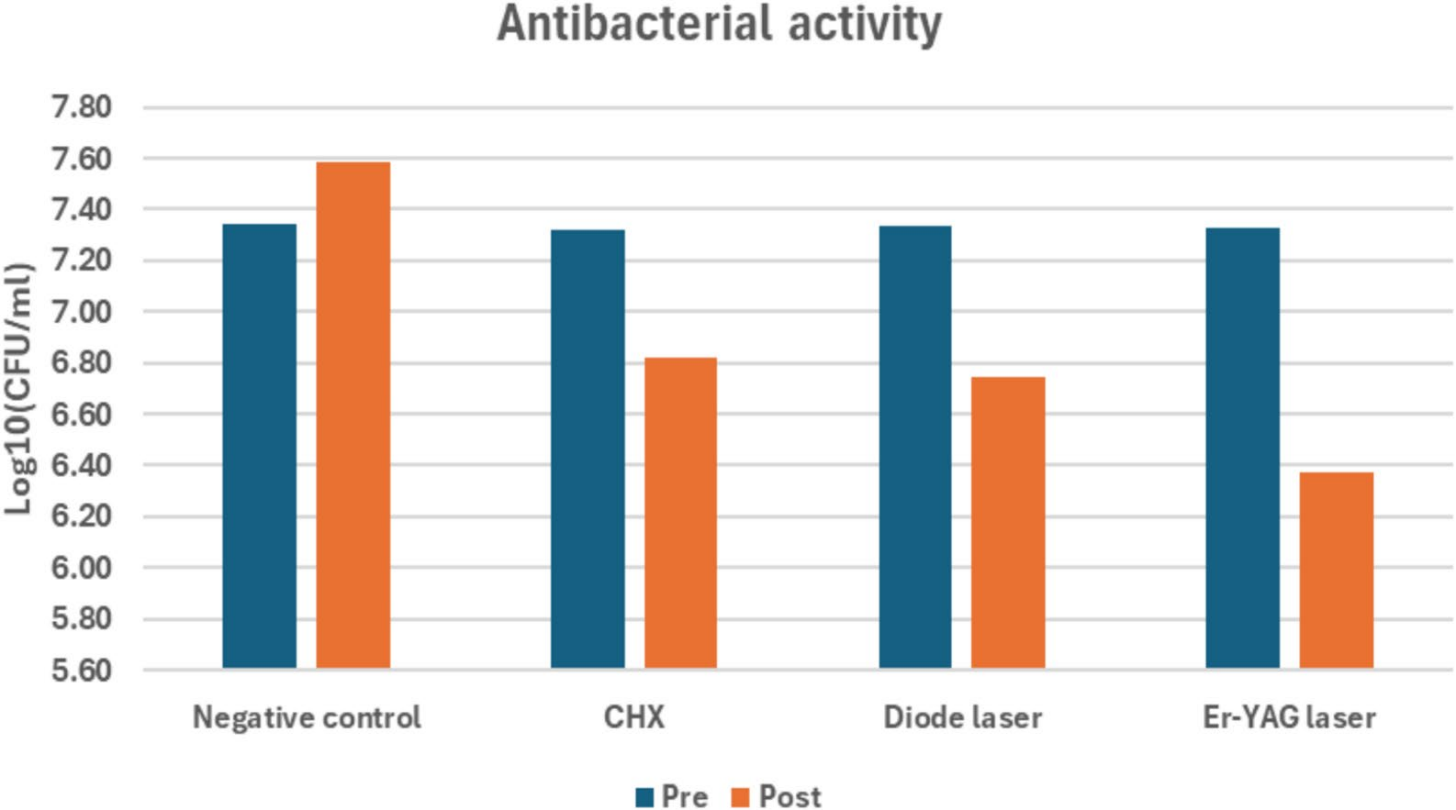
Bar chart representing antibacterial activity for different groups. Post-treatment comparison indicated a highly significant difference among groups (*p* < 0.001). The Er:YAG laser showed the lowest bacterial count, significantly lower than both CHX and diode laser (*p* = 0.002 and *p* = 0.012)

**Fig. 7:**
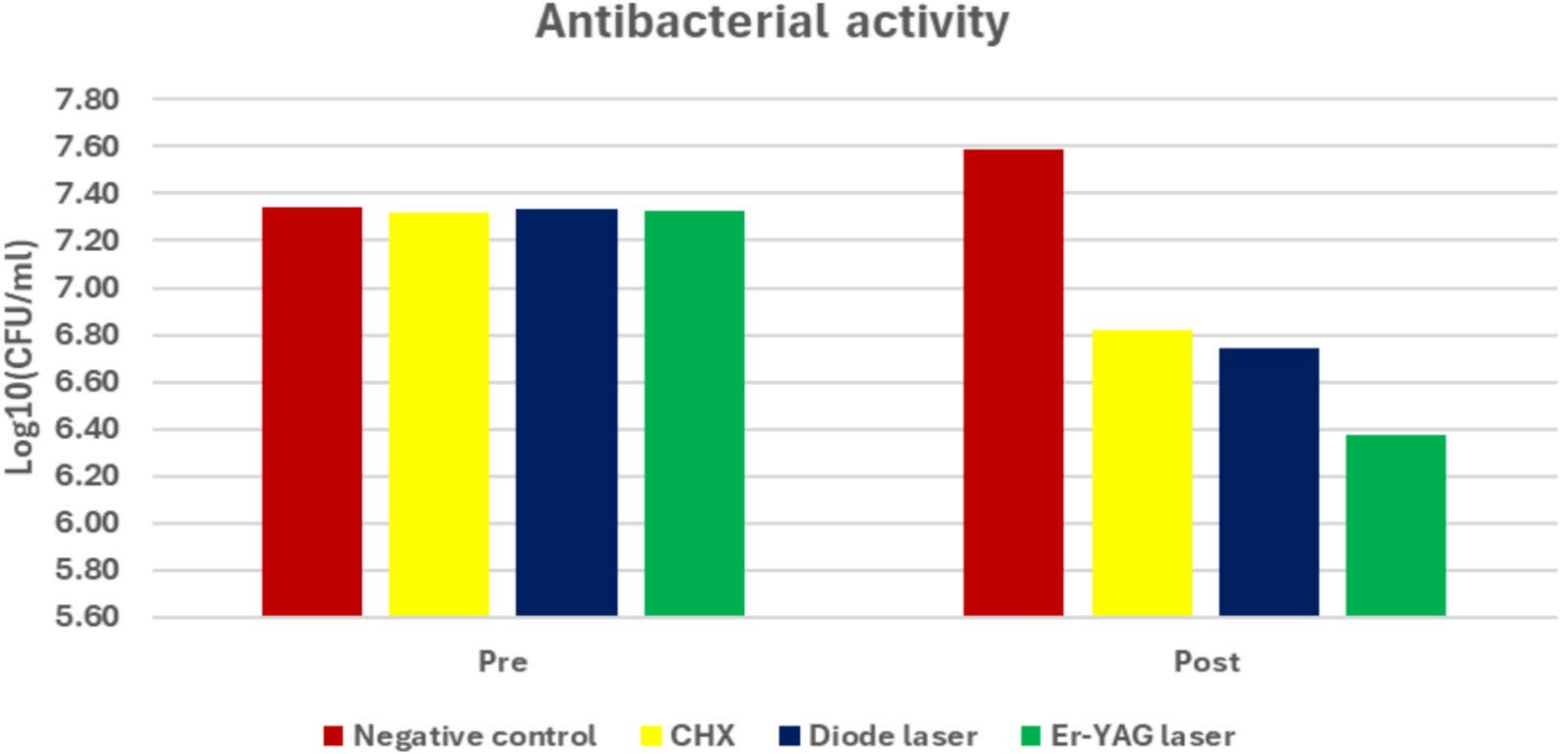
Bar chart representing antibacterial activity for different groups. Percentage of change in CFU mirrored OD results: Er:YAG = 12.99% ± 2.13, CHX = 6.90% ± 3.91, Diode = 8.02% ± 5.03, while the negative control showed bacterial increase (+ 3.31% ± 0.89)

**Fig. 8:**
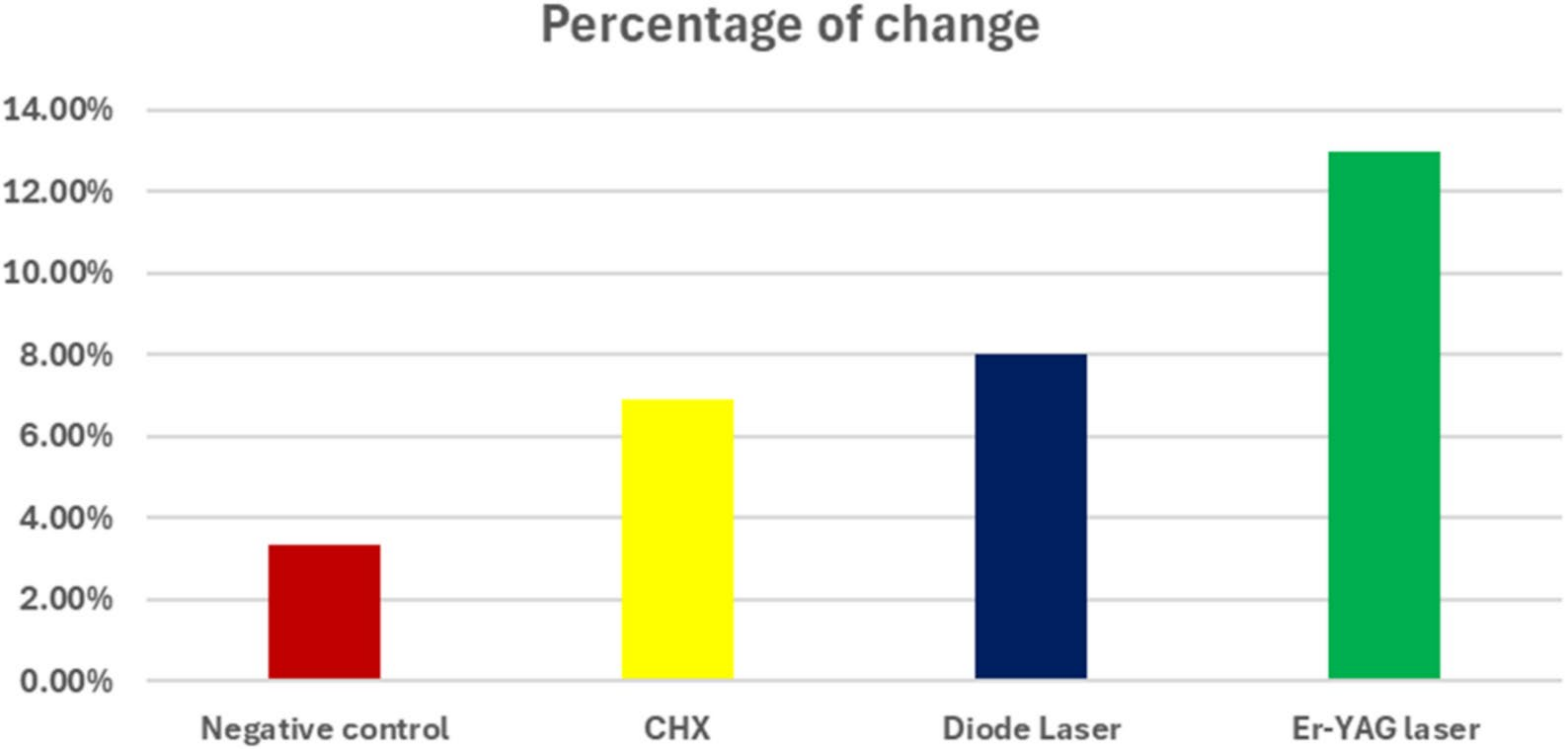
Bar chart representing percentage of bacterial change in different groups

To quantify the viable count of *Streptococcus mutans*, serial dilutions of each sample were prepared up to three dilution levels: 10⁻^1^, 10⁻^2^, and 10⁻^3^, using sterile saline as the diluent. From each dilution, 100 µL was aseptically pipetted and spread evenly onto Columbia blood agar plates using a sterile glass spreader to ensure uniform distribution. The plates were incubated anaerobically at 37 °C for 48 h to allow optimal growth of *S. mutans*. Following incubation, colonies were counted on plates that exhibited 30–300 colonies, and the number of CFUs per milliliter was calculated by multiplying the number of colonies by the dilution factor (Fig. 6, 7, 8) [12, 26]**.** Both Optical density and CFU Measurements were performed in two stages: first, as a baseline reading (pre-treatment) to determine the initial bacterial load, and then post-treatment, after application of the disinfection protocol under investigation.

Successful biofilm formation was verified indirectly through the statistically significant increase in bacterial load in the negative-control group following incubation, confirming the viability and activity of *S. mutans* biofilm prior to treatment.

#### Treatment modalities

In Group 2, the chlorhexidine group (positive control), disinfection was performed using a 2% chlorhexidine gluconate solution (Grace for dental industries, Egypt) applied at a flow rate of 10 mL/min for 60 s. Following application, the cavity was rinsed with sterile saline and air-dried to remove any residual solution. In Group 3, the diode laser group, a 980 nm diode laser (LASOTRONIX, Poland) was employed at a power setting of 1 W in continuous mode. The cavity surface was irradiated for a total of 60 s, divided into two 30-s cycles. A 8 mm tip was used, positioned 1 mm from the cavity in a perpendicular orientation, and moved in a scanning motion to ensure even coverage., spot size 8 mm (as shown in supplementary Fig. 1) Group 4, the Er:YAG laser group, was treated using a 2940 nm Er:YAG laser (Fotona: AT Fidelies Ljubljana, Slovenia) with settings, including a pulse energy of 120 mJ, frequency of 10 Hz, power output of 1.2 W, water and air levels set to 4, and SP (short pulse) mode with a pulse duration of 300 ms. The irradiation was delivered using an R02 tipless handpiece spot size 0.9 mm for a total of 60 s, divided into two 30-s cycles(as shown in supplementary Fig. 2).

The power of Er:YAG laser was selected according to a pilot study conducted before the beginning of the main study. The range between 50 and 150 mj is considered safe regarding dentine morphology and mineralization [22] the pilot study was done using 50, 100, 120, 150 mj. Both 120 and 150 mj energy showed the highest antibacterial efficiency against *Streptococcus mutans* bacteria with no statistically significant difference between them; so the energy of Er:YAG laser chosen was 120 mj.

#### Post-treatment incubation

Following treatment, samples were incubated for another 48 h under the same conditions to assess residual bacterial viability after different treatment modalities

#### Bacterial growth assessment

Bacterial growth assessment was evaluated by measuring the optical density (OD) of the culture medium using a NanoStar ELISA microplate reader tables (1, 2) and colony forming units (CFU) measurement in the same manner that was previously mentioned 

**Table 1:** Mean, standard deviation (SD) values of bacterial count of different groups

Variables	Antibacterial activity				
	Pre		Post		*p* value
	Mean	SD	Mean	SD	
Negative control	0.318	0.044	0.556	0.076	< 0.001*
CHX	0.300	0.017	0.119	0.015	< 0.001*
Diode laser	0.307	0.020	0.126	0.015	< 0.001*
Er–YAG laser	0.305	0.024	0.050	0.011	< 0.001*
*p* value	**0.554 ns**		< 0.001*		

^*^significant (p < 0.05) *ns* non-significant (p > 0.05)

**Table 2:** Mean, standard deviation (SD) values of percentage of bacterial change of different groups

Variables	Antibacterial activity Percentage of change	
	Mean	SD
Negative control	**42.298%**	**8.085**
CHX	**60.225%**	**6.571**
Diode laser	**58.542%**	**6.840**
Er–YAG laser	**83.500%**	**4.079**
*p* value	< 0.001*	

^*^significant (*p* < 0.05)

## Statistical analysis

Mean and standard deviation values were calculated for each group under all test conditions. The data distribution was assessed for normality using the Kolmogorov–Smirnov and Shapiro–Wilk tests, confirming a parametric (normal) distribution. For comparisons involving more than two independent groups, one-way analysis of variance (ANOVA) was applied, followed by Tukey’s post hoc test for pairwise comparisons. The paired sample test was employed to analyze differences between two related groups. A significant level of *p* ≤ 0.05 was adopted for all statistical tests. Data analysis was conducted using IBM^®^ SPSS^®^ Statistics software, version 20 (IBM Corp., Armonk, NY, USA).

## Results

### Antibacterial effect based on optical density (OD) measurements

All groups showed comparable baseline bacterial loads before treatment (*p *= 0.554). The negative control group demonstrated a significant increase in bacterial growth after incubation (*p* < 0.001), confirming bacterial proliferation without disinfection.

Chlorhexidine (CHX) and diode laser treatment both produced significant bacterial reductions (p < 0.001), with mean post-treatment OD values of 0.119 ± 0.015 and 0.126 ± 0.015, respectively. The Er:YAG laser group showed the greatest bacterial reduction from 0.305 ± 0.024 to 0.050 ± 0.011 (*p* < 0.001).

### Antibacterial effect based on colony-forming units (CFU/mL)

Baseline CFU counts were statistically comparable among all groups (*p* = 0.671). The negative control group showed bacterial growth from 7.34 ± 0.06 to 7.59 ± 0.06 log10 CFU/mL (*p* < 0.001). All disinfectant-treated groups showed significant bacterial reductions (*p* < 0.001): CHX (7.32 ± 0.02 → 6.82 ± 0.29), diode laser (7.33 ± 0.03 → 6.75 ± 0.38), and Er:YAG laser (7.33 ± 0.03 → 6.38 ± 0.15). tables (3, 4)

**Table 3:** Mean, standard deviation (SD) values of Log10 (CFU/ml) of different groups

Variables	Antibacterial activity				
	Pre		Post		*p* value
	Mean	SD	Mean	SD	
Negative control	**7.34**	**0.06**	**7.59**	**0.06**	< 0.001*
CHX	**7.32**	**0.02**	**6.82**	**0.29**	< 0.001*
Diode laser	**7.33**	**0.03**	**6.75**	**0.38**	< 0.001*
Er–YAG laser	**7.33**	**0.03**	**6.38**	**0.15**	< 0.001*
*p* value	**0.671 ns**		< 0.001*		

^*^significant (*p* < 0.05) *ns* non-significant (*p* > 0.05)

**Table 4:** Mean, standard deviation (SD) values of percentage of bacterial change of different groups

Variables	Antibacterial activity Percentage of change	
	Mean	SD
Negative control	**3.31%**	**0.89**
CHX	**6.90%**	**3.91**
Diode laser	**8.02%**	**5.03**
Er–YAG laser	**12.99%**	**2.13**
*p* value	< 0.001*	

^*^significant (*p* < 0.05)

### Summary of findings

Both optical density and CFU analyses demonstrated consistent trends

- All disinfection methods significantly reduced bacterial counts compared with the control.
- Er:YAG laser exhibited the highest antibacterial efficacy, followed by CHX and diode laser, with no statistical difference between the latter two.
- The agreement between OD and CFU results confirms the reproducibility and reliability of the findings.

## Discussion

Recurrent caries is a condition that can emerge in conjunction with pre-existing restoration and accounts for a significant portion of restorative replacement in primary and permanent teeth. The clinical problem of recurring caries applies to all dental restorations, regardless of chemical structure or placement technique. Recurrent caries has been documented to be one of the most common causes of restorative failure [27].

*Streptococcus mutans* (*S. mutans*) are consistently associated with caries initiation and progression under filling materials developing recurrent caries, because they have a unique ability to thrive under environmental stress conditions, particularly low pH (aciduricity) and a long-term lack of carbohydrates [4, 28].

Cariogenic bacteria can live in the smear layer and dentin tubules during cavity preparation, so all contaminated tissues must be removed to ensure the final restoration's longevity. Otherwise, secondary caries can arise, resulting in restorative failure. Different cavity disinfectants have been recommended by researchers, such as different chemical agents, laser, ozone, and others [2, 29].

Chlorhexidine is regarded as the gold standard antibacterial agent in dentistry due to its potent antibacterial properties, which arise from the Chlorhexidine (CHX) molecule’s capacity to disrupt the bacterial cell membrane, enhancing permeability and inducing cell lysis. [8]. It is well-established that it can lead to staining and discoloration of tooth surfaces and may have cytotoxic effects on human fibroblasts by inhibiting protein synthesis [1, 30].

This study utilized a 2% chlorhexidine solution, as it is the most effective concentration for cavity disinfection, and its liquid form enhances penetration into the dentinal tubules [8]. Because of its capacity to effectively remove any residual viable germs, the diode laser is widely used in the disinfection of dental cavities and periodontal pockets, as well as in endodontic treatment [7, 31]. This antimicrobial impact is related to the temperature increase that induces bacterial cell lysis; characterized as the photo thermal effect [32]. In the current study, cavity disinfection was performed using a diode laser 980 nm with an output power of 1 W, since it is a safe parameter that has previously been utilized in cavity disinfection without producing any thermal damage to the pulp [33].

Erbium:YAG laser can lower bacterial load, remove the surface dentin layer, and easily offer access to mechanically unreachable parts of the dentinal tube network, as well as exert additional antibacterial effects [34]. In earlier investigations, various authors revealed considerable bacterial reductions in the diseased dentin that can be possibly sterilized by Er:YAG laser light through its transmission properties by its photothermal and photodisruptive effects [20, 35].

Er:YAG laser with 2.94 µm is shown to have bactericidal effects on *S. mutans*, We used a low energy output (1.2 Watt) as it is regarded to be safe to avoid any possible harmful effects of temperature rise in dentin, and moreover, the reduction of the dentin hardness, as according to Du et al., the power range between 1 and 1.5 was found to be efficient against *Streptococcus mutans* bacteria without causing any harmful effects to dentine [36].

Microbiological assessment of *Streptococcus mutans* bacteria was performed in the current study, as they are the main bacteria responsible for producing dental caries in experimental animals and humans [37, 38]. ELISA technique was used in this study which is regarded as an effective and accurate approach for identifying *Streptococcus mutans* bacteria [39].

Microbiological assessment was done in this study via Optical density and colony forming units' methods. Optical density is widely used for estimating bacterial growth due to its rapid, non-destructive nature, making it ideal for real time monitoring in routine microbiological studies [40], even though OD measurements do not directly indicate the number of viable bacteria cells, to obtain biologically meaningful data, OD values must be calibrated with colony forming unit (CFU) counts to convert optical measurements into meaningful biological data. Despite the limitation of OD measurements, it remains a cornerstone of bacterial enumeration [25].

All tested disinfectant groups (CHX, diode laser, and Er:YAG laser) demonstrated statistically significant reductions in bacterial load when comparing post-treatment values to baseline. The CHX group decreased from 0.300 ± 0.017 to 0.119 ± 0.015, the diode laser group decreased from 0.307 ± 0.020 to 0.126 ± 0.015, and the Er:YAG laser group showed the greatest reduction, decreasing from 0.305 ± 0.024 to 0.050 ± 0.011 (all p < 0.001). In contrast, the negative control group demonstrated a statistically significant increase in bacterial count, rising from 0.318 ± 0.044 to 0.556 ± 0.076 after incubation (p < 0.001), indicating active bacterial proliferation in the absence of a disinfectant [41]. These numerical outcomes confirm the effectiveness of cavity disinfection using CHX and laser-based approaches, and highlight that intervention is necessary to prevent bacterial growth within the prepared cavity [8, 20, 36].

A statistically significant difference was found between the Er:YAG laser and CHX groups, with the lowest post-treatment bacterial count observed in the Er:YAG group (0.050 ± 0.011) compared with the CHX group (0.119 ± 0.015). This finding aligns with previous studies reporting that Er:YAG laser irradiation has a highly efficient antimicrobial effect. Its antibacterial action is attributed not only to direct bacterial elimination, but also to the morphological and chemical alterations induced on dentin [14]. At higher energy densities, Er:YAG irradiation may cause superficial melting of the dentin surface, which has been associated with enhanced antibacterial performance and a smoother surface that may reduce recolonization and lower the likelihood of secondary caries [36]. Conversely, Sancakli et al. reported that CHX exhibited superior antimicrobial activity compared with Er:YAG when used as a cavity disinfectant; however, this discrepancy may be related to methodological differences, as their study employed a lower laser power (1 W) and shorter exposure duration (5 s) [20].

A statistically significant difference was found between the Er:YAG laser and diode laser groups, with the lowest post-treatment bacterial count recorded in the Er:YAG group (0.050 ± 0.011) compared with the diode laser group (0.126 ± 0.015). To the best of our knowledge, no previous study has directly compared the antibacterial performance of Er:YAG laser and diode laser as cavity disinfectants. However, the superior effect observed with Er:YAG in the present study may be attributed to the high absorption of Er:YAG irradiation at 2940 nm by dentin, together with its ability to remove the smear layer which is known to harbor residual bacteria in addition to its intrinsic antimicrobial action [34].

Furthermore, even when operated at sub-ablative settings, the Er:YAG laser can induce surface micro-morphological alterations in dentin. These changes include surface smoothing, which results in fewer surface irregularities capable of retaining bacterial deposits [42].

Our results demonstrated that both CHX and the 980-nm diode laser produced strong antibacterial effects against *Streptococcus mutans*, with no statistically significant difference between the two modalities. The CHX group decreased from (0.300 ± 0.017 to 0.119 ± 0.015), while the diode laser group decreased from (0.307 ± 0.020 to 0.126 ± 0.015), and both reductions were statistically significant (p < 0.001). This finding is consistent with AbdelHamid et al. [8]**,** who similarly reported comparable antibacterial performance between CHX and diode laser disinfection. However, other studies reported different outcomes. For example, Vinothkumar et al. found that the diode laser produced greater antibacterial reduction than CHX; this inconsistency may be attributed to differences in diode laser wavelength, as their investigation used an 810 nm wavelength—unlike our 980 nm parameter—despite applying a similar power setting (1 W) [26].

## Conclusion

Within the limitations of this in-vitro study, Er:YAG laser irradiation demonstrated the highest antibacterial efficacy against *Streptococcus mutans* bacteria, significantly outperforming both CHX and diode laser disinfection. These findings suggest that Er:YAG laser may represent a more effective cavity disinfection modality prior to adhesive procedures and restoration placement.

## Limitation of study

This research was conducted under laboratory conditions, which may not fully replicate the clinical environment; therefore, further in vivo studies are required to validate these findings. The study focused on the short-term antibacterial effect (48 h) and did not evaluate long-term outcomes. Moreover, only a quantitative assessment of bacterial reduction was performed, without a qualitative evaluation. Consequently, future studies incorporating microscopic or molecular analyses are recommended to provide deeper insight into bacterial alterations and structural effects.

## Supplementary Information

Supplementary material 1.

## Data Availability

The datasets used and/or analyzed during the current study are available from the corresponding author upon reasonable request. All efforts were made to avoid compromising an individual's privacy.
