# Supplementary material for: The antibacterial effect of chlorohexidine, Er:YAG laser and diode laser 980 nm as dental cavity disinfectants of dentine: an in vitro study
Source: Eur J Med Res. 2025 Dec 29;30:1284. doi: 10.1186/s40001-025-03595-z (PMC12751903; doi:10.1186/s40001-025-03595-z)

**Supplementary Figure 1 illustrates the application of the diode laser.**


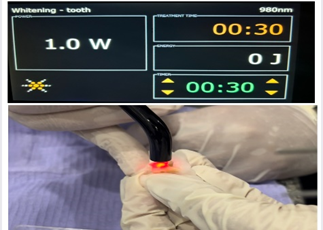


**Supplementary Figure 2 illustrates the application of the Er;YAG laser.**


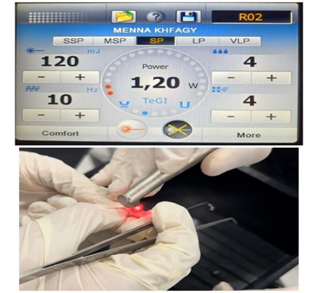

Supplement: Supplementary file 1 — Supplementary material 1. [file 40001_2025_3595_MOESM1_ESM.docx]
